# Preoperative Oral Carbohydrate (CHO) Supplementation Is Beneficial for Clinical and Biochemical Outcomes in Patients Undergoing Elective Cesarean Delivery under Spinal Anaesthesia—A Randomized Controlled Trial

**DOI:** 10.3390/jcm12154978

**Published:** 2023-07-28

**Authors:** Katarzyna Kotfis, Arleta Wojciechowska, Małgorzata Zimny, Dominika Jamioł-Milc, Aleksandra Szylińska, Sebastian Kwiatkowski, Karolina Kaim, Barbara Dołęgowska, Ewa Stachowska, Maciej Zukowski, Maria Pankowiak, Andrzej Torbé, Paul Wischmeyer

**Affiliations:** 1 Department of Anesthesiology, Intensive Therapy and Acute Intoxications, Pomeranian Medical University in Szczecin, 70-111 Szczecin, Poland; kaim.karolinna@gmail.com (K.K.); maciej.zukowski@pum.edu.pl (M.Z.); maria.pankowiak@gmail.com (M.P.); 2 Department of Obstetrics and Gynecology, Pomeranian Medical University in Szczecin, 70-111 Szczecin, Poland; arletawoj@gmail.com (A.W.); mmzimny@wp.pl (M.Z.); sebastian.kwiatkowski@pum.edu.pl (S.K.); andrzej.torbe@pum.edu.pl (A.T.); 3 Department of Human Nutrition and Metabolomics, Pomeranian Medical University, 71-460 Szczecin, Poland; dominika.jamiol.milc@pum.edu.pl (D.J.-M.); ewa.stachowska@pum.edu.pl (E.S.); 4 Department of Medical Rehabilitation and Clinical Physiotherapy, Pomeranian Medical University in Szczecin, 71-210 Szczecin, Poland; aleksandra.szylinska@gmail.com; 5 Department of Microbiology, Immunology and Laboratory Medicine, Pomeranian Medical University in Szczecin, 70-111 Szczecin, Poland; barbara.dolegowska@pum.edu.pl; 6 Department of Anesthesiology and Surgery, Duke University School of Medicine, Durham, NC 27710, USA; paul.wischmeyer@duke.edu

**Keywords:** nausea, vomiting, PONV, ERAS, fasting, obstetrics, cesarean section, OCH, oral carbohydrate, F2-isoprostane, outcomes

## Abstract

Background: Preoperative fasting and surgery cause metabolic stress, insulin resistance with ketosis, and postoperative nausea and vomiting (PONV). Oral carbohydrate loading strategy (CHO) improves outcomes in labor and general surgery. We aimed to compare the effectiveness of CHO with standard fasting in patients undergoing elective cesarean delivery (CD) under spinal anesthesia. Methods: A single-center, parallel, prospective randomized controlled trial (RCT) was conducted in a tertiary university obstetrics department at Pomeranian Medical University in Szczecin, Poland. Patients were randomly assigned (1:1 ratio) to the CHO group (oral carbohydrate 2 h before elective CD, *n* = 75) or the SF group (control—standard fasting, *n* = 73). The main outcome measures were incidence and severity of PONV at 6 and 24 h after CD, time to the first peristalsis, time to first bowel movement, and biochemical parameters indicating ketosis in mothers and their children. Results: A total of 148 adult females with singleton pregnancies undergoing elective CD under spinal anesthesia (ASA I and II) were included in the final analysis. At 24 h after CD, 8.0% from the CHO group vs. 20.55% reported three or more episodes of vomiting or dry retching as compared to patients in the SF group (*p* = 0.041). Preoperative CHO supplementation decreased preoperative feelings of hunger (*p* < 0.001) and thirst (*p* < 0.001). Laboratory results in the CHO group showed higher plasma pH (*p* = 0.001) and glucose (*p* < 0.001), lower F2-isoprostane in plasma (*p* = 0.049) and urine (*p* = 0.018), lower urine F2-isoprostane/creatinine ratio (*p* = 0.045) than in the SF group. HOMA-IR (*p* < 0.001) and lactate (*p* < 0.001) were higher in the CHO group than in the control group. Conclusions: There was no significant difference in the incidence or severity of early PONV at 6 h. The incidence of vomiting or dry retching at 24 h after cesarean delivery was lower in the CHO group as compared to standard starvation, but the combined results of PONV frequency and severity on the Wengritzky scale did not differ between the two study groups. Preoperative CHO supplementation decreased preoperative feelings of hunger and thirst, enhancing the comfort of pregnant women. Trial registration: ClinicalTrials.gov identifier: NCT04069806.

## 1. Introduction

Post-operative and intraoperative nausea and vomiting (PONV/IONV) is an important clinical morbidity in women undergoing subarachnoid anesthesia for cesarean delivery (CD) that occurs in up to 80% of patients [1,2]. The mechanisms underlying PONV are associated with prolonged fasting, hypotension due to vasodilation during spinal anesthesia, bradycardia due to increased vagal tone, visceral stimulation during surgery, anesthetic technique, and intrathecal administration of opioids [1,3,4]. Perioperative fasting has traditionally been utilized to prevent vomiting and as a means of reducing perioperative mortality associated with aspiration pneumonia [5,6,7,8,9]. This is essential to address as fasting is uncomfortable for the patient and may lead to dehydration without a clear reduction in the incidence of aspiration, can cause metabolic stress, ketosis, abnormal serum pH, mitochondrial dysfunction, hyperlactatemia, Beta-OH-butyrate and isoprostane elevation, hypoglycemia, decreases hepatic glycogen stores, induces insulin resistance, and causes impaired cardiovascular function [10,11,12,13].

The European Society of Anaesthesiology and Intensive Care (ESAIC) Guideline recommends that adults and children should be encouraged to drink clear fluids up to 2 h before elective surgery (including cesarean delivery) with solid foods prohibited for 6 h before elective surgery; contemporary perioperative guidelines for cesarean delivery reflect this approach (ERAS guidelines based on ESAIC and ASA recommendations) [9,14,15,16,17,18]. The major problem, however, lies not in the recommended 6- and 2-hour timeframes, but in the fact that due to organizational issues, these fasting times are regularly exceeded before surgery [6,9,19].

It has been shown that oral carbohydrate (CHO) intake may improve outcomes in general and cardiac surgery [20] and in labor [21,22,23,24]; therefore, it may be extrapolated that oral carbohydrate loading may attenuate hypotension associated with vasodilation during spinal anesthesia and thereby reduce associated nausea and vomiting. Although women in many obstetric wards receive a carbohydrate drink before elective cesarean delivery, as recommended by the current guidelines, the metabolic effects of these drinks on the mother and neonate have not been fully evaluated. According to the Guidelines for Antenatal and Preoperative care in Cesarean Delivery: Enhanced Recovery After Surgery Society Recommendations, an oral carbohydrate fluid supplementation may be offered to nondiabetic pregnant women 2 h before cesarean delivery; however, the evidence level was low and the recommendation level graded as weak [17]. However, there is research that confirms the significance of the proposed intervention on the metabolic effect [25,26]. These recommendations are based on studies in adult general surgery (gastrointestinal, gynecology, oncology) [27] and in obstetrics in labor [17,24].

The working hypothesis for this study was that in patients undergoing elective cesarean delivery under spinal anesthesia, oral carbohydrate loading strategy as compared with standard pre-operative fasting is superior regarding the incidence and severity of postoperative nausea and vomiting (IONV/PONV), time to the first peristalsis, time to first bowel movement, and biochemical parameters indicating ketosis.

## 2. Materials and Methods

### 2.1. Study Design

We conducted a single-center, parallel, prospective randomized controlled trial (RCT).

### 2.2. Study Setting

The study was conducted between August 2019 and March 2020 in the Department of Obstetrics and Gynecology, at Pomeranian Medical University in Szczecin, Poland. This research project was carried out according to the current version of the Declaration of Helsinki (amendment 2013) by the World Medical Association and the ICH-GCP Guidelines E6(R2) and the current Declaration of Istanbul. The study received approval from the Bioethical Committee of the Pomeranian Medical University in Szczecin, Poland, approval no. KB-0012/113/19, issued on 13 May 2019. In all cases, every eligible patient willing to participate in the study signed the informed consent form before inclusion in the study and initiation of study procedures. The study was prospectively registered before the inclusion of the first patient at ClinicalTrials.gov with Identifier: NCT04069806.

### 2.3. Patients

The study group included a total of 154 adult pregnant women. The inclusion criteria for the study were: age ≥ 18 years, singleton gestation at term (37–42 weeks), uncomplicated pregnancy, patients scheduled for an elective cesarean delivery, planned for spinal anesthesia, and ASA I or ASA II.

The following exclusion criteria were applied: (1) preexisting diabetes (gestational diabetes, type I DM, type II DM); (2) history of gastro-intestinal reflux (GERD); (3) history of bariatric surgery; (4) pre-pregnancy BMI > 40; (5) unable or unwilling to sign an informed consent; (6) contraindications to spinal anesthesia; (7) contraindication to oral carbohydrate formula.

### 2.4. Interventions

#### 2.4.1. Randomization

We used a computer-generated randomization table (generated by the primary investigator before study initiation from the randomizer.org website) to allocate the patients into two groups in a 1:1 ratio. A member of the team not performing any other tasks was responsible for the patient allocation; therefore, after assignment to one of the two groups, the participants, care providers, and personnel assessing outcomes were blinded.

Group I (*n* = 75) included patients randomized to receive oral carbohydrate drinks (CHO) and Group II (*n* = 73) included patients randomized to serve as controls and receive standard fasting (SF). Women in Group I (CHO) received a standard oral carbohydrate drink (200 mL) with 12.5% dextrose in water (Pre-op^®^, N.V. Nutricia, Zoetermeer, The Netherlands) in addition to standard fasting (advised for 6 h for solids and 2 h for clear liquids). Women in Group II (SF) were advised to fast for 6 h for solids and 2 h for clear liquids as per local protocol before elective cesarean delivery.

#### 2.4.2. Perioperative Care

All participants were scheduled for a cesarean delivery under spinal anesthesia. All patients received 15 mL of sodium citrate and an appropriate antibiotic prophylaxis infusion (most commonly cefazoline) within 30 min before surgery as per local protocol. After the placement of an intravenous line, a 500 mL volume of a balanced crystalloid infusion was initiated. Spinal anesthesia was performed with the use of 0.5% hyperbaric bupivacaine with 200 micrograms of preservative-free morphine and the dose titrated according to patient height as per the discretion of the anesthetist (between 2.2 and 3.5 mL of total volume). After achieving a satisfactory level of sensory block tested via cold sensation (T4 level, bilaterally), a urinary catheter was placed, and the cesarean delivery was performed. The cesarean delivery procedure was performed according to a local protocol with standard Cohen incision and subcuticular skin closure; exteriorization of the uterus was avoided. Treatment of hypotension with intravenous ephedrine and the volume of fluids infused during cesarean delivery was performed at the discretion of the anesthetic team, composed of experienced consultants and senior residents. The postoperative care was performed in the anesthetic recovery room for a minimum of 30 min and in the postoperative obstetric ward thereafter. The postoperative pain control regime included 1 g of paracetamol every 6 h orally or intravenously, ibuprofen 400 mg every 6 h orally, and oxycodone 5 mg orally if postoperative pain exceeded 4 points out of 10 on the numeric rating scale (NRS).

### 2.5. Data Collection

#### 2.5.1. Demographic and Perioperative Data

##### Maternal Data

Data were collected using a special questionnaire with the following sections: (1) data from the patient interview (demography data, vomiting in early pregnancy, previous anesthesia, motion sickness), (2) pre-operative data (length of fasting, volume of fluid therapy), (3) perioperative data (doses of drugs for anesthesia, level of subarachnoid blockade, final level of anesthesia), and (4) postoperative data (intensity of pain, first peristalsis, first bowel movement).

##### Neonatal Data

The data regarding the well-being of the newborn were recorded from medical records, including one-minute and five-minute Apgar scores and birth weight recorded at delivery. To monitor neonatal outcomes, umbilical cord blood was drawn for blood glucose, lactate, and pH.

### 2.6. Biochemical Analysis

Ketosis parameters were evaluated in both the mother and the newborn to compare the influence of fasting versus oral carbohydrate loading. The study procedure included a collection of a total of 7.5 mL of maternal venous blood (collected during intravenous routine venipuncture for venous cannula insertion before cesarean delivery, for intravenous fluid and drug administration) and collection of 10 mL of urine obtained after catheterization with a Foley catheter before CD (routine procedure as per local protocol). The biochemical parameters included: serum pH, glucose, lactate, and Beta-OH-butyrate in the mother’s venous blood and creatinine and 15-F(2t)-isoprostane level in the urine obtained before the cesarean delivery.

Serum glucose level was evaluated using the enzymatic method (Cobas 8000 analyzer, Roche, Poland). The parameters of pH, lactate, and glucose concentration were determined using a POCT analyzer (GEM Premier 3500, Werfen, Bedford, MA, USA). The concentration of Beta-OH-butyrate was determined by a spectrophotometric method using a ready-made reagent kit (Pointe Scientific Polska, Warszaw, Poland). The concentration of isoprostane was determined by the ELISA method using the 8-isoprostane ELISA Kit (Cayman Chemical, Ann Arbor, MI, USA).

The data regarding the well-being of the newborn were recorded from medical records and biochemical parameters. These data included values obtained from a blood gas from the umbilical cord and as part of routine testing performed on the newborn. Neonatal pH, glucose, and lactate levels were measured in the whole blood (GEM Premier 3500, Werfen, Bedford, MA, USA).

### 2.7. Main Outcome Measures

#### Study Endpoints

The primary outcome measures were postoperative nausea and vomiting (PONV) intensity at 6 and 24 h after cesarean delivery. To assess the postoperative nausea and vomiting (PONV) intensity, the Wengritzky Scale was used (Scale range: 0–50. The scale measures PONV intensity, where a score of ≥50 is defined as clinically important PONV: PONV intensity scale = severity of nausea (1 = mild, 2 = moderate, 3 = severe) × pattern of nausea (1 = varying, 2 = constant) × duration of nausea (in hours)).

The secondary outcome measures were: (1) Time to the first peristalsis after the operation. (2) Time to first bowel movement after the operation. (3) Concentration of maternal serum lactate. (4) Concentration of maternal Beta-hydroxy-butyric acid in serum. (5) Concentration of glucose level measured in maternal serum. (6) Insulin resistance factor (HOMA-IR) measured in maternal serum. (7) Concentration of 15-F(2t)-isoprostane in maternal urine. (8) Concentration of maternal serum lactate. (9) Concentration of neonatal glucose level.

### 2.8. Additional Assessments

Changes between the maternal groups were assessed by the mean arterial pressure (MAP) recordings every 3 min. Patient satisfaction was recorded at baseline and after 24 h using a well-being numeric rate scale. The number of days in the hospital was recorded for each participant and compared between the groups. Neonatal plasma glucose level was evaluated and neonates with a plasma glucose level of less than 45 mg/dL were considered hypoglycemic. Neonatal well-being was assessed with an Apgar score evaluated at minutes 1, 5, and 10 after birth. There were no changes to trial outcomes after the trial commenced. There were no interim analyses.

### 2.9. Statistical Analysis

All data were analyzed using the software Statistica 13 (StatSoft, Inc., Tulsa, OK, USA). Categorical variables were presented as proportions and compared using the chi-square test; in the case of small numbers in groups, the Yates correction was applied. Continuous variables are presented as means with standard deviation and medians and first quartile and third quartile. Differences in baseline characteristics of patients with and without the use of CHO were compared using the Mann–Whitney U test or Student’s *t*-test. Statistical significance was set at a value of *p* < 0.05. We calculated a priori that a total of 154 (77 per group) would be needed to detect a reduction in the incidence of PONV from 50% to 25% in the CHO group with 90% power and significance level of 5%, allowing for 25% loss to follow-up (i.e., emergency deliveries before the planned date of surgery).

## 3. Results

### 3.1. Study Group Characteristics

**Figure 1:**
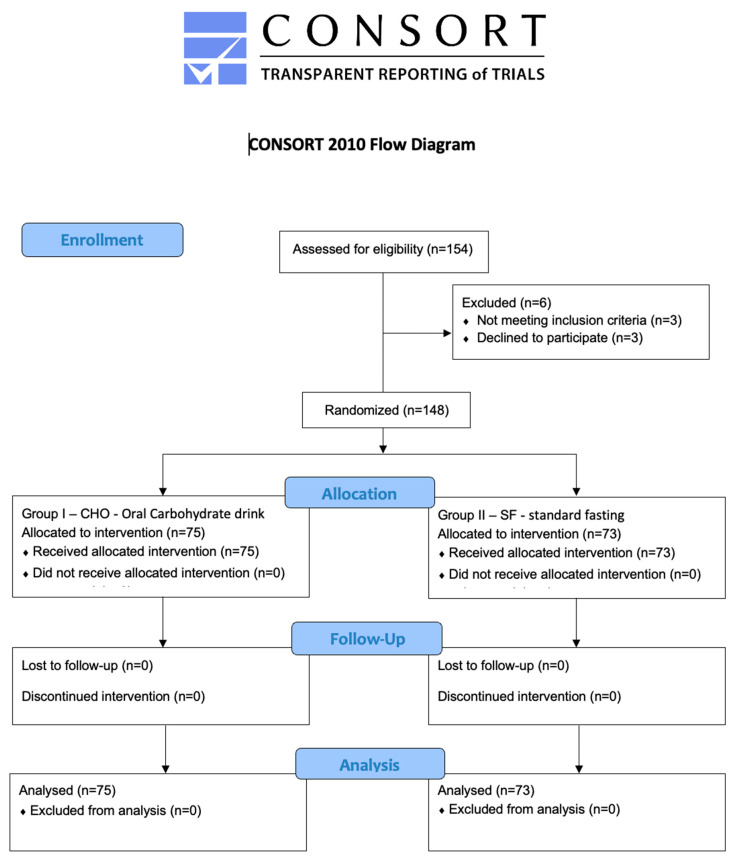
CONSORT flow diagram of the progress through stages of a randomized controlled trial of two parallel groups.

Between 1 August 2019 and 31 March 2020, in total, 154 patients were assessed for eligibility and 148 patients entered the study (3 did not meet inclusion criteria—additional information was revealed by the patient after signing informed consent and 3 patients declined to participate) as seen in Figure 1. The study was stopped after a pre-defined number of patients entered the study. There were no patients lost to follow-up.

The average age of the patients was 31.6 years. The average height of the examined women was 165.86 cm, while the recorded average weight was 78.28 kg. The mean body mass index (BMI) was 28.42. Table 1 shows the demographic parameters and medical conditions of the included patients, no statistically significant differences were found between both groups. The comparison of the two groups regarding medications taken by patients for concomitant diseases did not reveal any statistically significant differences.

Supplementary Table S1 presents risk factors predisposing to PONV. The results of the patients in both studied groups did not differ statistically significantly.

### 3.2. Intraoperative Factors

In the assessment of perioperative factors, no statistically significant differences were found between the study and control groups (Table 2).

Supplementary Table S2 shows the change in mean arterial pressure (MAP) during the cesarean delivery from baseline (time T0) and assessments performed at minute 1 (T1), minute 4 (T4), and minute 7 (T7) and after completion of the cesarean delivery (Tfinal) versus baseline values before cesarean delivery, i.e., after the onset of anesthesia. There was a statistically significant difference between the two groups and the most profound MAP decrease at 7 min after the initiation of spinal anesthetic.

### 3.3. Primary Outcome Measures

The analysis of the severity of nausea and vomiting according to the Wengritzky scale at 6 and 24 h after cesarean delivery is presented in Table 3. At 6 h, in the CHO group, 28/75 (37.3%) and in the SF group 20/73 (27.4%) developed vomiting (*p* = 0.196) and 38/75 (50.7%) in the CHO group complained of nausea versus 35/73 (47.9%) in the SF group (*p* = 0.741). After 6 h after CD, the duration of nausea in the control group was 0.25 h vs. 0.57 h in the CHO group (*p* = 0.339). At 24 h, in the CHO group 23/75 (30.67%) and in the SF group 24/73 (32.9%) developed vomiting (*p* = 0.773), whereas 30/75 (40%) in the CHO group and 36/73 (49.3%) in the SF group complained of nausea (*p* = 0.285). However, at 24 h after cesarean delivery, significantly fewer patients from the CHO group reported three or more episodes of vomiting or dry retching compared to patients in the standard fasting group (8.0% vs. 20.55%, *p* = 0.041). The combined results of PONV frequency and severity did not differ between the two groups (0.20 (0.00–10.00) vs. 0.30 (0.00–9.00), *p* < 0.574).

This scale measures PONV intensity, where a score ≥ 50 is defined as clinically important PONV.

Wengritzky scale:

Q1—Have you vomited or had dry-retching: 0—no; 2—once or twice; 50—three or more times.

Q2—Have you experienced a feeling of nausea: 0—no; 1—sometimes; 2—often or most of the time; 25—all the time.

Q3—Has your nausea been mostly: 1—varying (“comes and goes”); 2—constant (“is nearly or almost always present”).

Q4—What was the duration of your feeling of nausea (in hours (whole or fraction))?

### 3.4. Secondary Outcome Measures

#### Biochemical Parameters

Another aim of the study was to evaluate the biochemical parameters between patients receiving CHO and SF. The results are presented in Supplementary Table S3.

**Figure 2:**
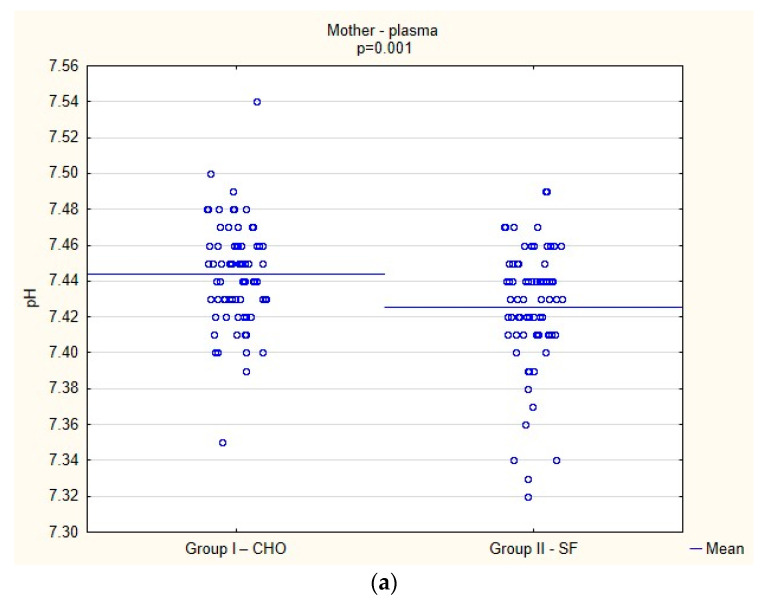

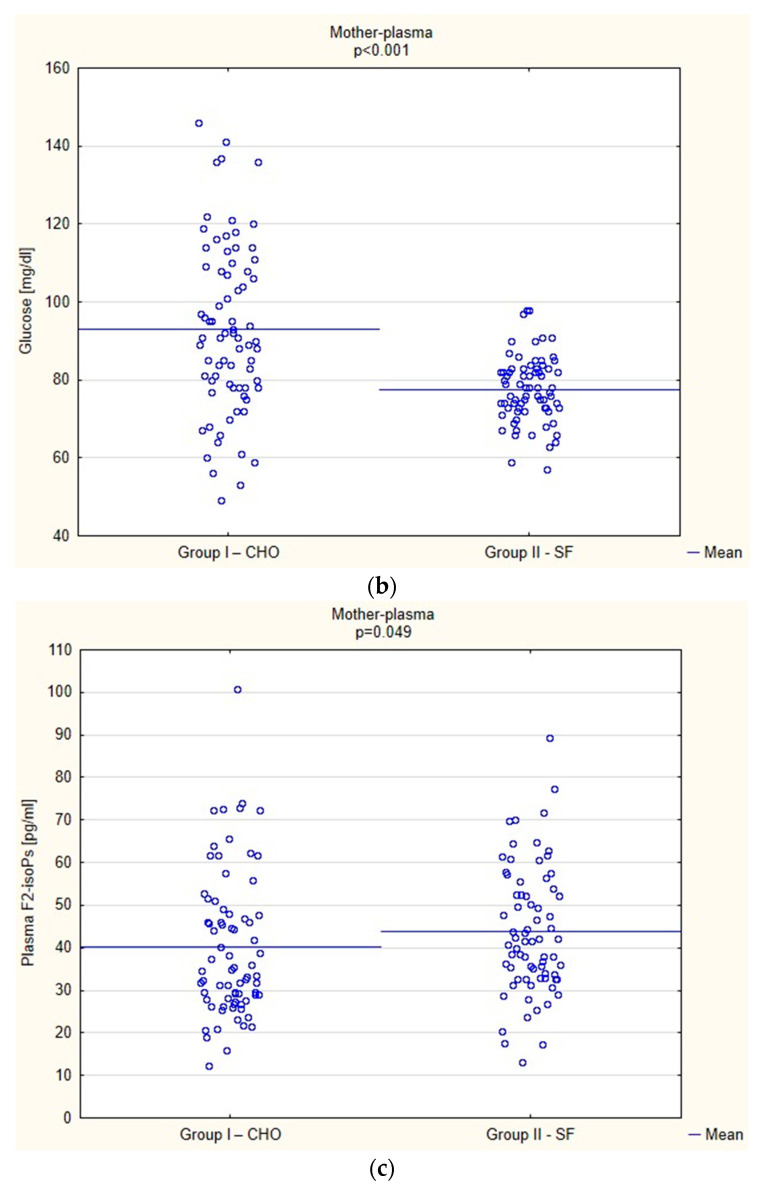

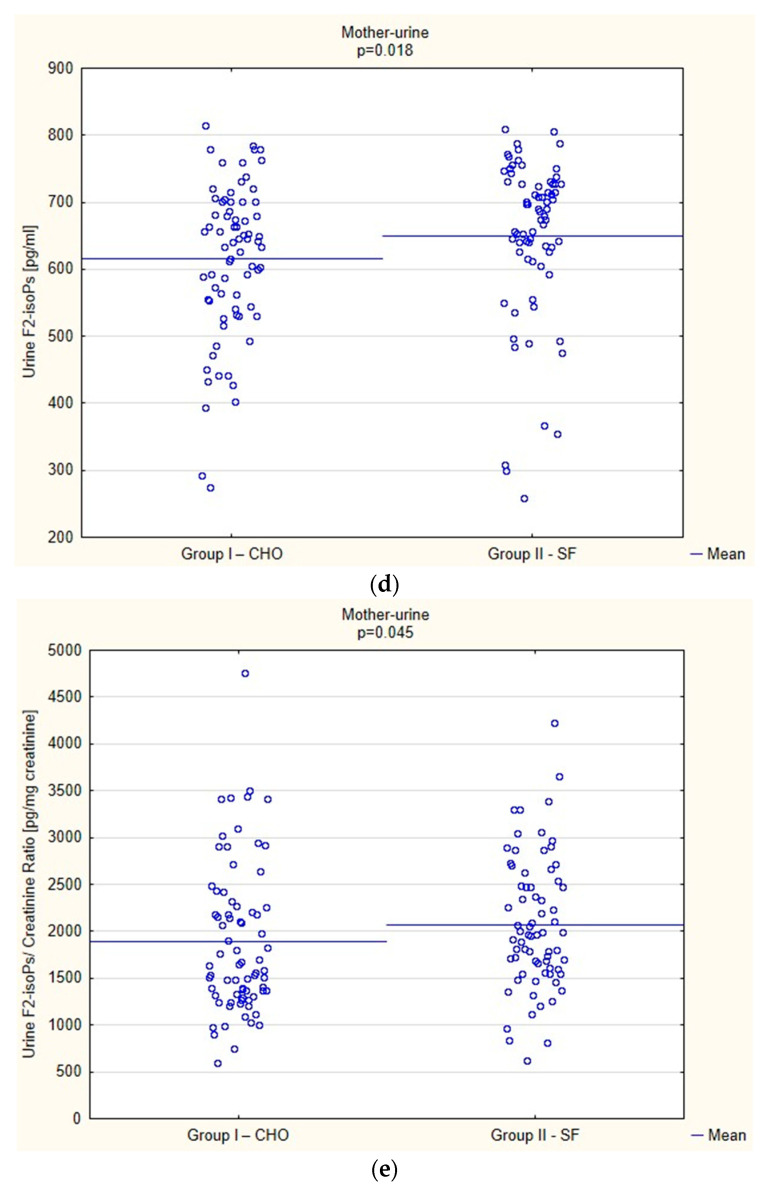

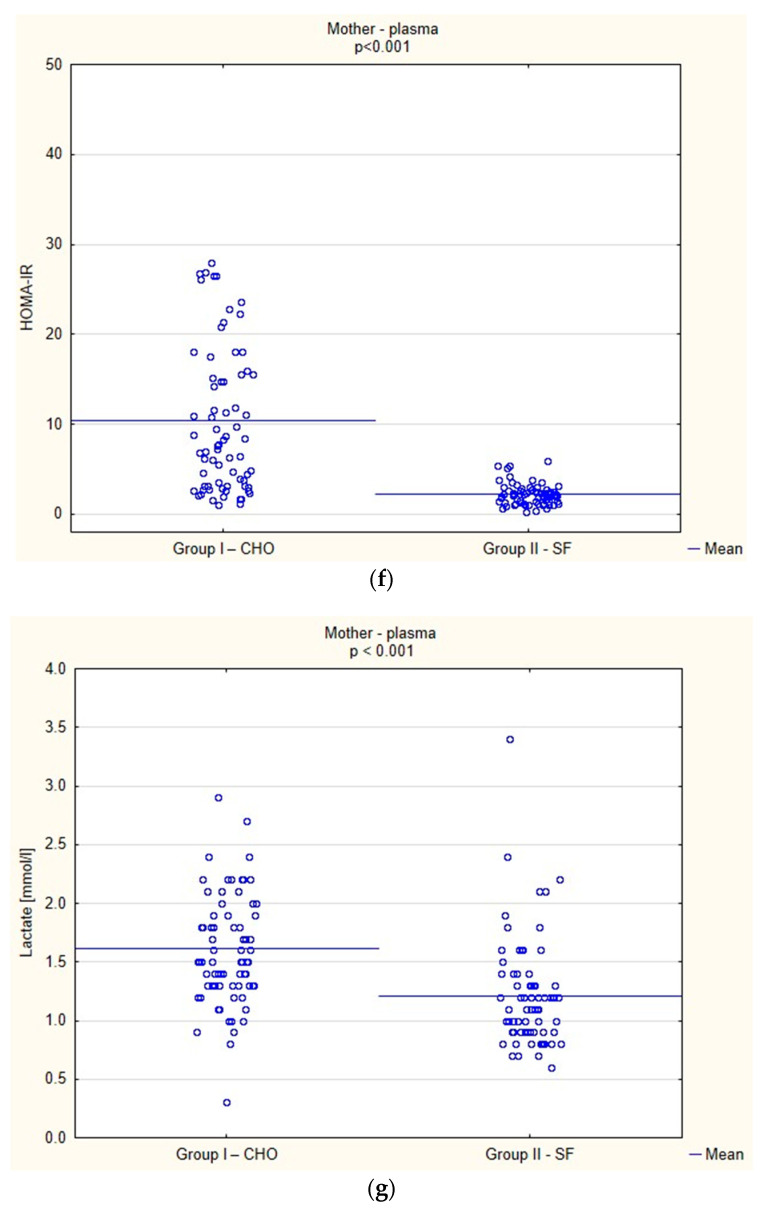

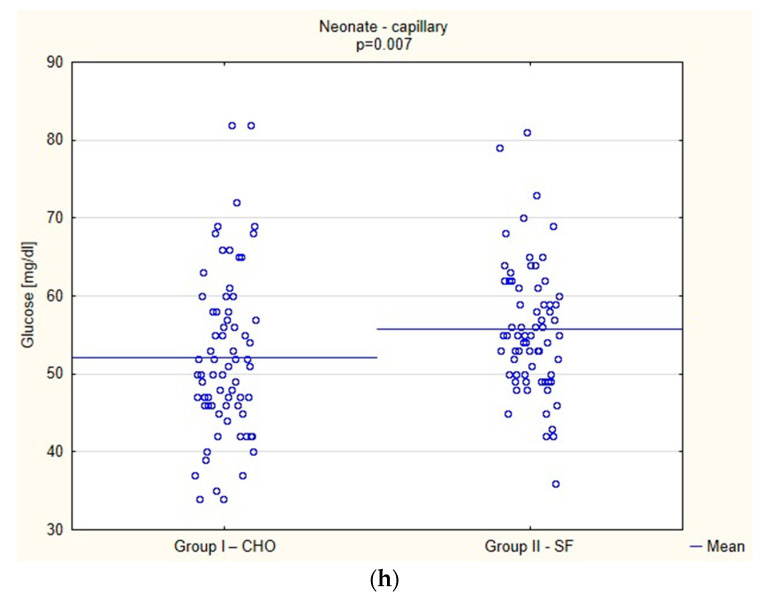
(**a**) The pH level in maternal plasma. (**b**) The fasting glucose level in maternal plasma. The ketosis marker, i.e., the level of F2-isoprostane, was significantly lower in patients receiving CHO both in plasma (40.15 ± 16.89 vs. 43.78 14.91; *p* = 0.049) and in urine (615.42 ± 115.11 vs. 649.97 ± 121.41; *p* = 0.018), as was the urine F2-isoPs/creatinine ratio (1.896.12 ± 797.12 vs. 2.073.46 ± 701.84; *p* = 0.045) (**c**–**e**). (**c**) The F2-isoPs level in maternal plasma. (**d**) The F2-isoPs level in maternal urine. (**e**) The F2-isoPs/creatinine ratio in maternal urine. Interestingly, the insulin resistance index, HOMA-IR, was statistically significantly higher in the CHO group (10.45 ± 8.94 vs. 2.19 ± 1.15, *p* < 0.001) than in the control group (**f**), as was the serum lactate level in the study group (1.62 ± 0.49 mmol/L vs. 1.21 ± 0.46 mmol/L, *p* < 0.001) compared to the control group as shown in (**g**), although statistically different does not seem to be clinically significant. (**f**) HOMA-IR in maternal plasma. (**g**) Lactate level in maternal plasma. Quite contrary to mothers’ glucose levels, the analysis of neonatal data showed that in the group of mothers who received CHO, the level of glucose in the newborn’s capillary blood was lower, than in the newborns whose mothers did not receive CHO (52.10 mg/dL vs. 55.76 mg/dL, *p* = 0.007). (**h**) The glucose level in neonatal capillary blood.

The analysis of the laboratory results is shown in Figure 2a–h. Higher plasma pH (*p* = 0.001) (Figure 2a) and glucose (*p* < 0.001) values (Figure 2b) were shown in the group of patients who received CHO as compared to the control group.

### 3.5. Clinical Parameters

The evaluation of postoperative factors for mothers and newborns is presented in Table 4. In patients who received CHO, the time without eating food was longer than in patients from the control group (10.31 h vs. 9.13 h, *p* = 0.035). On the other hand, the time without consuming drinks was shorter in the CHO group (6.62 h vs. 14.32 h, *p* < 0.001). The time to first peristalsis (10.92 ± 5.67 h vs. 12.41 ± 6.49, *p* = 0.213) did not differ between the two subgroups. In both groups, the first bowel movement occurred later than 72 h after the operation (94.67% vs. 94.52%, *p* = 0.746).

The birth weight of newborns was higher in the group of patients who received CHO (*p* = 0.033). In the study group, no newborn required additional administration of oral glucose, whereas in the control group, six newborns required additional administration of oral glucose as ordered at the discretion of the attending neonatologist on duty (*p* = 0.039). Please note that the hypoglycemia reported here comes from the measurement from the neonatal blood right after birth and the data regarding additional glucose use mean any need of glucose administration recorded during the first 24 h post-partum. Therefore, it is very probable that the initial hypoglycemia resolved immediately with breastfeeding.

### 3.6. Patient Satisfaction

The subjective feeling of hunger (*p* < 0.001) and thirst (*p* < 0.001) before cesarean delivery was significantly lower in the group of patients who received CHO (Supplementary Table S4), but no statistically significant differences were found 6 h afterward.

There was no harm or unintended effect in any of the groups.

## 4. Discussion

The primary objective of this randomized controlled study was to evaluate the effect of oral carbohydrate drinks as compared with standard pre-operative fasting on the incidence and severity of postoperative nausea and vomiting in patients undergoing elective cesarean delivery under spinal anesthesia. As compared to other published studies in this area, our analysis includes the largest number of enrolled patients and reports the most comprehensive clinical and biochemical data regarding both the mother and the neonate.

The study showed no significant difference in the incidence or severity of early PONV at 6 h after cesarean delivery in the CHO group as compared to standard starvation. The use of a CHO drink resulted in fewer patients from the CHO group reporting three or more episodes of vomiting or dry retching at 24 h post-cesarean delivery (a score > 50 on the Wengritzky scale) compared to patients in the standard fasting group (8.0% vs. 20.55%, *p* = 0.041), but the experienced feeling of nausea, the duration of feeling of nausea, and the combined results of PONV frequency and severity did not differ between the two study groups. Preoperative CHO supplementation decreased preoperative feelings of hunger (*p* < 0.001) and thirst (*p* < 0.001). The ketosis markers (F2-isoprostane) were significantly lower in patients receiving CHO both in plasma and in urine as was the urine F2-isoPs/creatinine ratio.

Spinal anesthesia for cesarean delivery may induce perioperative hypotension, yet in our study, the perioperative hemodynamic data showed no significant differences between the two groups; thus, this component did not add to the risk of IONV or PONV. Since we did not detect a clinically significant difference regarding blood pressure between the two groups, our hypothesis regarding the attenuating effect of carbohydrate loading before cesarean delivery could not be demonstrated. Therefore, possible reasons for increased PONV for fasting parturients is associated with factors not related to blood pressure decrease.

Toohill et al. have shown that lactate and Beta-OH-butyrate in the mother’s venous blood and isoprostane level in the urine were elevated during fasting before CD [13]. Isoprostanes (prostaglandin F_2_-like compounds) have proven useful in assessing oxidative stress and lipid peroxidation during disease and fasting in experimental animal models and clinical practice [24,27,28]. The 15-F(2t)-isoprostane level may be used as a sensitive biomarker of fetal oxidative stress during labor that may be associated with ketosis [29].

Bellwood et al. examined a group of women before planned cesarean delivery to examine the factual time of fasting for mothers who were undergoing elective caesarean sections and the frequency of ketone bodies in urine before and after administration of the Nutricia PreOp 400 mL carbohydrate drink prior to surgery. The authors revealed that the average fasting time since the last calorie intake decreased from 13 h 35 min to 5 h 5 min after the pre-operative carbohydrate drink was administered. They also found that the frequency of ketone bodies in the urine was 40% prior to the introduction of the preoperative carbohydrate drink and 38% after the introduction of the preoperative drink (*p* = 1) [26].

Moreover, Clark et al. conducted a randomized trial to investigate the effect of the presence of ketone bodies in the urine of mothers undergoing elective cesarean delivery compared with standard care. The study involved 184 women who were divided into two groups: 90 patients received standard care and 94 were given a carbohydrate drink before the surgery. The frequency of ketone bodies in urine directly before surgery was lower in the carbohydrate-treated group, 18.1% compared with 61.1% in the standard care group (*p* < 0.001) [25].

Our results are consistent with data obtained in previous smaller studies with more limited sample sizes. According to Wendling et al. who recruited low-risk women undergoing scheduled cesarean deliveries with planned spinal anesthesia, either a common oral rehydration beverage or a higher-dose carbohydrate beverage consumed preoperatively results in superior well-being compared to fasting. No other differences in outcomes, including cord blood glucose level, intraoperative variables, breastfeeding success, and quality of recovery, were noted [10].

Shi et al. performed a randomized controlled pilot study aimed at evaluating the effect of preoperative oral carbohydrate administration on patients undergoing CD with epidural anesthesia [30]. A total of 75 patients undergoing CD (ASA I-II) were randomized to preparation with a carbohydrate drink (CHO group), flavored water (placebo group), or the fasting group. During the preoperative period, administration of CHO reduced not only thirst and anxiety more efficiently than water (placebo) but also hunger, whereas water did not. Compared with the preoperative levels, insulin resistance showed a statistically significant increase in all groups (*p* < 0.05). However, the increase was significantly higher in the fasting and placebo groups than in the CHO group (*p* < 0.05). Interestingly, in our study, the insulin resistance index (HOMA-IR ratio) was significantly higher in the CHO group as compared with the control group (*p* < 0.001). In contrast, a study by Shi et al. showed that insulin resistance (compared with the preoperative levels) showed a statistically significant increase in both the CHO and fasting groups (*p* < 0.05), but the increase was significantly higher in the fasting and placebo groups than in the CHO group (*p* < 0.05) [30].

A recent study by He et al., which included 88 pregnant women undergoing elective cesarean section randomized to receive oral carbohydrate drink or placebo, demonstrated that the CHO group had lower postoperative insulin levels and HOMA-IR index compared with women who had fasted [31]. Additionally, neonates of mothers who were allocated to the CHO group had higher glucose levels as compared with neonates of mothers in the fasting group [31]. The authors concluded that the comfort provided by improved preoperative thirst and hunger is higher in patients receiving oral carbohydrate loading as compared to fasted subjects. Preoperative CHO loading has been shown to reduce insulin resistance and decrease the time to the return of intestinal function compared to other clear liquids and fasting, without causing increased gastric acidity or volume [32].

Quite interestingly, our study showed that the insulin resistance index, HOMA-IR, was statistically significantly higher in the CHO group. We cannot find a definite answer to the fact that insulin resistance increased in this group as compared to the control group. Insulin resistance is a complex metabolic disorder associated with attenuated responsiveness of peripheral tissues (muscle, liver, adipose tissue) to insulin signaling; therefore, insulin release is increased to maintain glucose homeostasis [33]. IR is associated not only with type 2 diabetes or other metabolic disorders (metabolic syndrome, cardiovascular disease, obesity), but may also increase in non-diabetic patients [34]. Moreover, it was shown that even in the absence of diabetes or other metabolic disorders both inflammatory mechanisms and pro-inflammatory mediators (tumor necrosis factor-alpha or interleukin-6) have been involved in the pathogenesis of IR when pro-inflammatory cytokines develop inflammation through reactive oxygen species (ROS) and oxidative stress pathways [35]. This may induce IR in peripheral tissues and adipocytes, especially in obesity [36]. Yet, in our current study, the 8-isoprostane levels that were used as oxidative stress markers were lower in the CHO group, but the IR was higher. Our findings may indicate that other oxidative stress markers, unmeasured by us, including tumor necrosis factor-alpha or interleukin-6, could have been associated with IR increase in this group. Moreover, gestational weight gain is associated with decreased pancreatic beta-cell function and impaired glucose-insulin metabolism in overweight or obese pregnant women, although IR can also develop in individuals with normal body weight [37]. IR may also be linked with insulin action on protein and lipid metabolism or vascular endothelial function and gene expression [38].

Moreover, oral CHO consumption improves the sense of patient well-being as compared with fasted controls. Pain as well as nausea and vomiting are amongst the most common causes of readmission to the hospital in many surgical non-obstetric circumstances [39]. Spinal anesthesia with intrathecal opioids used for obstetric patients provides good analgesia but may lead to persistent PONV; therefore, further effort should be undertaken to limit this complication to improve the quality of perioperative care and reduce patient suffering.

This study also assessed data regarding newborns and breastfeeding. The birth weight of newborns in the group of patients who received CHO was higher (*p* < 0.05). No newborns in the CHO group received oral glucose, whereas in the control group six newborns received oral glucose due to hypoglycemia (*p* < 0.05) as per the decision of the attending neonatologist—the difference in management may have been related to the duration of hypoglycemia, normalization of the repeated measurement, and/or the timing of breastfeeding. Hypoglycemia reported in this study comes from the measurement from the neonate right after birth and the data regarding additional glucose use meant any need of glucose administration recorded during the first 24 h post-partum. Therefore, it is very probable that the initial hypoglycemia resolved immediately with breastfeeding. These results are key as one of the most common challenges of cesarean delivery is the delay in initiation of breastfeeding, which is due to pain, anxiety, stress, thirst, and hunger. A study conducted by Fard et al. evaluated the effect of preoperative oral carbohydrates on breastfeeding after cesarean delivery. In this double-blind randomized clinical trial, 91 pregnant women who underwent elective cesarean delivery were randomly assigned to preoperative CHO (Nutricia Pre-op^®^) or control group (water flavored with lemon). According to Fard et al., the time to first breastfeeding after surgery was significantly shorter in the CHO group than in the control group (*p* < 0.001), the frequency of breastfeeding was significantly higher (*p* < 0.001), and the mean duration of breastfeeding was significantly longer in the CHO during the first 36 h after the surgery (*p* < 0.001) [40].

Administration of oral carbohydrate loading in patients with diabetes mellitus, including pregnant women, has not been officially licensed, yet may prove to be reasonable and safe [41]. Currently, the use of carbohydrate loading preoperatively in type II diabetes has been an area of controversy and is often not utilized in patients with diabetes mellitus, especially in pregnancy. Although more research is needed, a recent review of existing data for perioperative CHO loading in type II diabetes shows that CHO loading does not appear to be associated with an increased risk of aspiration or significant hyperglycemia and may improve outcomes such as reducing the length of stay [42]. Furthermore, the preoperative use of carbohydrate loading in nonpregnant patients with diabetes mellitus was recently evaluated in a prospective, noninferiority cohort study. These data revealed that CHO drink provision was non-inferior to fasting, and neither group showed superiority for preoperative blood glucose concentration, hyperglycemia, or length of stay [42]. The analysis of our laboratory results showed that those patients who received CHO had higher plasma glucose (*p* < 0.001) values as compared to the control group, yet within normal limits. According to Liu et al., perioperative CHO drink administration was also safe for patients with gestational diabetes mellitus (GDM) undergoing cesarean delivery [43], although the levels of blood glucose and serum insulin right before induction of anesthesia were significantly higher in the CHO group than those in the control group, hunger scores were lower and no aspiration, nausea, or vomiting occurred in either group before, during, and after surgery.

### Limitations

This study is not without limitations. This is a monocentric study; therefore, its generalizability may be limited. To address this, the authors plan to perform a multi-center RCT utilizing the same study protocol. The sources of potential bias include a lack of placebo control, which is difficult to overcome—the use of artificial sweeteners may lead to biochemical changes.

## 5. Conclusions

The study showed no significant difference in the incidence or severity of early PONV at 6 h. The incidence of vomiting or dry retching at 24 h after cesarean delivery was lower in the CHO group as compared to standard starvation, but the combined results of PONV frequency and severity on the Wengritzky scale did not differ between the two study groups. The ketosis markers were significantly lower in patients receiving CHO in plasma and urine. No significant difference was found in the time to the first flatus and first abdominal movement between the two groups. Preoperative CHO supplementation decreased preoperative feelings of hunger and thirst by the patients, enhancing the comfort of pregnant women. CHO also positively affects the neonate as no newborns required additional administration of oral glucose due to hypoglycemia.

## Figures and Tables

**Table 1:** Demographic parameters.

Data	Group I—CHO (*n* = 75)		Group II—SF (*n* = 73)	
Demographic data				
Age, Me, Q1–Q3	31.00	28.00–36.00	34.00	29.00–36.00
Height, Me, Q1–Q3	167.0	161.00–170.00	165.00	160.00–170.00
Weight, Me, Q1–Q3	77.0	69.50–84.0	78.00	72.00–85.00
BMI, Me, Q1–Q3	28.04	25.51–30.12	28.65	26.47–31.22
Parity, Me, Q1–Q3	2.0	1.00–2.00	2.00	2.00–3.00
Birth, Me, Q1–Q3	2.0	1.00–2.00	2.00	1.00–2.00
Pregnancy week, Me, Q1–Q3	39.0	39.00–40.00	39.00	39.00–39.00
Pre-pregnancy medical history				
Hypertension, *n* (%)	1	(1.33%)	1	(1.37%)
Valvular disease, *n* (%)	0	(0.00%)	1	(1.37%)
Thrombosis, *n* (%)	1	(1.33%)	1	(1.37%)
Thrombophilia, *n* (%)	2	(2.67%)	0	(0.00%)
Varicose veins, *n* (%)	1	(1.33%)	2	(2.74%)
Hypothyroidism, *n* (%)	11	(14.67%)	12	(16.44%)
Hyperthyroidism, *n* (%)	1	(1.33%)	0	(0.00%)
Hashimoto disease, *n* (%)	2	(2.67%)	2	(2.74%)
Multiple sclerosis, *n* (%)	1	(1.33%)	0	(0.00%)
Epilepsy, *n* (%)	0	(0.00%)	1	(1.33%)
Asthma, *n* (%)	2	(2.67%)	2	(2.74%)
Medical problems in pregnancy				
None, *n* (%)	54	(72.00%)	36	(49.32%)
Hypertension	2	(2.67%)	5	(6.85%)
Hypothyroidism	8	(10.67%)	11	(15.07%)
Infection	2	(2.67%)	6	(8.22%)
Other	3	(4.00%)	9	(12.33%)
Obstetric	6	(8.00%)	6	(8.22%)
Medications				
Methyldopa, *n* (%)	5	(6.67%)	5	(6.85%)
Metoprolol, *n* (%)	0	(0.00%)	1	(1.37%)
Enoxaparin, *n* (%)	3	(4.00%)	3	(4.17%)
Levothyroxine, *n* (%)	19	(25.33%)	23	(31.51%)
Levetiracetam, *n* (%)	1	(1.33%)	0	(0.00%)
Salbutamol, *n* (%)	2	(2.67%)	3	(4.11%)

Legend: BMI—body mass index, CHO—oral carbohydrate drink, Me—median, *n*—number of patients, Q1—first quartile, Q2—second quartile, Q3—third quartile, SF—standard fasting.

**Table 2:** Perioperative and anesthesia-related data.

Intraoperative Factors		Group I—CHO (*n* = 75)		Group II—SF (*n* = 73)		*p*-Value
Cesarean delivery duration time (min), Me, Q1–Q3		28.00	24.00–33.00	26.00	21.00–33.00	0.132
Anesthesia care duration time (min), Me, Q1–Q3		45.00	400.0–56.0	43.00	38.00–52.00	0.283
Marcaine 0.5% Spinal Heavy (mL), Me, Q1–Q3		2.08	2.60–3.00	2.90	2.80–3.20	0.314
Bupivacaine 0.5% hydrochloride Spinal Heavy (mL), Me, Q1–Q3		2.80	2.60–30.0	2.90	2.80–3.00	0.132
Morphine sulfate Spinal 0.1% (mcg), Me, Q1–Q3		200.0	200.0	200.0	200.0	0.998
Spinal blockade level prior to cesarean delivery (Th), *n* (%)	2	1	(1.33%)	0	(0.00%)	0.802
	3	1	(1.33%)	0	(0.00%)	
	4	21	(28.00%)	21	(28.77%)	
	5	8	(10.67%)	8	(10.96%)	
	6	37	(49.33%)	37	(50.68%)	
	7	7	(99.33%)	6	(8.22%)	
	8	0	(0.00%)	1	(1.37%)	
Spinal blockade level prior after cesarean delivery (Th), *n* (%)	2	15	(20.00%)	16	(21.92%)	0.563
	3	27	(36.00%)	29	(39.73%)	
	4	26	(34.67%)	21	(28.77%)	
	5	1	(1.33%)	4	(5.48%)	
	6	5	(6.67%)	3	(4.11%)	
	8	1	(1.33%)	0	(0.00%)	
Crystalloids *n* (%)	Optilyte	73	(97.33%)	73	(100.00%)	0.484
	Sterofundin	2	(2.67%)	0	(0.00%)	
Volume of crystalloids (mL), Me, Q1–Q3		1000.0	1000.00–1000.0	1000.0	1000.0–1000.0	0.135
Colloids (4% Gelaspan), *n* (%)	No	68	(90.67%)	68	(93.15%)	0.801
	Yes	7	(9.33%)	5	(6.85%)	
Volume of colloids (mL), Me, Q1–Q3		500.0	500.00–1000.0	500.0	500.0–500.0	0.508
ION, *n* (%)	No	47	(62.67%)	37	(50.68%)	0.192
	Yes	28	(37.33%)	36	(49.32%)	
IOV, *n* (%)	No	70	(93.33%)	70	(95.89%)	0.746
	Yes	5	(6.67%)	3	(4.11%)	
Antiemetics *n* (%)	No	67	(89.33%)	64	(87.67%)	0.936
	Yes	8	(10.67%)	9	(12.33%)	
	Ondansetron	5	(6.67%)	6	(8.22%)	
	Metoclopramide	3	(4.00%)	3	(4.11%)	
Ephedrine dose (mg), Me, Q1–Q3		20.0	10.00–25.00	20.00	10.00–25.00	0.872

Legend: CHO—oral carbohydrate drink, ION—intraoperative nausea, IOV—intraoperative vomiting, Me—median, Q1—first quartile, Q2—second quartile, Q3—third quartile, *n*—number of patients, SF—standard fasting, Th—thoracic dermatome level.

**Table 3:** Wengritzky scale at 6 and 24 h after cesarean delivery.

Wengritzky Scale		Group I—CHO (*n* = 75)	Group II—SF (*n* = 73)	*p*-Value
Nausea 6 h after cesarean delivery				
Q1 (6), *n* (%)	0 points	47 (62.67%)	53 (72.60%)	0.197
	2 points	16 (21.33%)	15 (20.55%)	
	50 points	12 (16.00%)	5 (6.85%)	
Q2 (6), *n* (%)	0 points	37 (49.33%)	38 (52.05%)	0.172
	1 point	26 (34.67%)	31 (42.47%)	
	2 points	10 (13.33%)	4 (5.48%)	
	25 points	2 (2.67%)	0 (0.00%)	
Q3 (6), *n* (%)	1 point	32 (84.21%)	34 (97.14%)	0.139
	2 points	6 (15.79%)	1 (2.86%)	
Q4 (6): (h) Me (Q1–Q3)		0.10 (0.00–0.40)	0.00 (0.00–0.10)	0.339
Result A (6), Me, (Q1–Q3)		0.10 (0.00–2.00)	0.00 (0.00–0.40)	0.252
Nausea 24 h after cesarean delivery				
Q1 (24), *n* (%)	0 points	52 (69.33%)	49 (67.12%)	0.041
	2 points	17 (22.67%)	9 (12.33%)	
	50 points	6 (8.00%)	15 (20.55%)	
Q2 (24), *n* (%)	0 points	44 (59.46%)	37 (50.68%)	0.369
	1 point	23 (31.08%)	25 (34.25%)	
	2 points	6 (8.11%)	11 (15.07%)	
	25 points	1 (1.35%)	0 (0.00%)	
Q3 (24), *n* (%)	1 point	26 (86.67%)	34 (94.44%)	0.506
	2 points	4 (13.33%)	2 (5.56%)	
Q4 (24): (h), Me, (Q1–Q3)		0.00 (0.00–0.15)	0.00 (0.00–0.30)	0.264
Result B (24), Me, (Q1–Q3)		0.00 (0.00–0.20)	0.00 (0.00–1.00)	0.196
Combined Result A + B, Me, (Q1–Q3)		0.20 (0.00–10.00)	0.30 (0.00–9.00)	0.574

Legend: CHO—oral carbohydrate drink, Me—median, Q1—first quartile, Q2—second quartile, Q3—third quartile, *n*—number of patients, SF—standard fasting.

**Table 4:** Clinical postoperative data—mother and neonate.

Postoperative Data—Mother		Group I—CHO (*n* = 75)		Group II—SF (*n* = 73)		*p*-Value
Withdrawal from drinking after CD (h), Me, Q1–Q3		3.00	2.30–3.70	3.00	2.50–4.00	0.614
Withdrawal from eating after CD (h), Me, Q1–Q3		9.20	7.30–12.00	8.00	5.50–11.0	0.035
Total withdrawal from drinking (h), Me, Q1–Q3		6.10	5.50–7.30	14.80	11.50–17.10	<0.001
Total withdrawal from eating (h), Me, Q1–Q3		23.20	21.00–26.60	23.20	19.70–25.70	0.568
Antiemetics after CD, *n* (%)	No	50	(66.67%)	48	(65.75%)	0.358
	Yes	25	(33.34%)	25	(34.25%)	
	Ondansetron	20	(26.67%)	23	(31.51%)	
	Metoclopramide	2	(2.67%)	2	(2.74%)	
	Both drugs	3	(4.00%)	0	(0.00%)	
Time to first peristalsis (h), Me, Q1–Q3		10.00	7.00–13.00	11.70	6.70–17.00	0.213
Time to first bowel movement (h), *n* (%)	<72 h	4	(5.33%)	4	(5.48%)	0.746
	>72 h	71	(94.67%)	69	(94.52%)	
Fluids iv, *n* (%)	No	6	(8.00%)	14	(19.18%)	0.077
	Yes	69	(92.00%)	59	(80.82%)	
	Crystalloids	69	(92.00%)	58	(79.45%)	
	Crystalloids and Colloids	0	(0.00%)	1	(1.37%)	
Volume of iv fluids (mL), Me, Q1–Q3		1000.0	500–1000.00	1000.0	500.0–1000.00	0.807
Postoperative data—neonate		Group I—CHO (*n* = 75)		Group II—SF (*n* = 73)		*p*
Time between spinal anesthesia and birth (min), Me, Q1–Q3		9.00	8.00–11.00	9.00	8.00–11.00	0.664
Gender, *n* (%)	female	30	(40.00%)	29	(39.73%)	0.973
	male	45	(60.00%)	44	(60.27%)	
Apgar I, Me, Q1–Q3		9.00	8.00–10.00	10.0	10.00–10.00	0.182
Apgar II, Me, Q1–Q3		10.00	10.00–10.00	10.0	10.00–10.00	0.264
Apgar III, Me, Q1–Q3		10.00	10.00–10.00	10.0	10.00–10.00	0.332
Birth weight (g), Me, Q1–Q3		3510.0	3200.0–3730.0	3350.0	3080.0–3640.0	0.033
Lowest weight after birth (g) Me, Q1–Q3		3240.0	3010.0–3480.0	3140.0	2795.0–3405.0	0.063
Decrease in body weight after birth (%), Me, Q1–Q3		7.63	6.37–8.90	7.26	6.13–8.26	0.163
Breastfeeding, *n* (%)	No	7	(9.33%)	2	(2.74%)	0.182
	Yes	68	(90.67%)	71	(97.26%)	
Additional feeding, Day I, *n* (%)	No	59	(78.67%)	52	(71.23%)	0.039
	MM	16	(21.33%)	15	(20.55%)	
	G	0	(0.00%)	6	(8.22%)	
Additional feeding, Day II, *n* (%)	No	47	(62.67%)	44	(60.27%)	0.665
	MM	25	(33.33%)	28	(38.36%)	
	G	2	(2.67%)	1	(1.37%)	
	BMM	1	(1.33%)	0	(0.00%)	

Legend: BMM—breastfeeding and modified milk, CD—cesarean delivery, CHO—oral carbohydrate drink, G—glucose, Me—median, Q1—first quartile, Q2—second quartile, Q3—third quartile, MM—modified milk, *n*—number of patients, SF—Standard fasting. Apgar I—at 1 min, Apgar II—at 5 min, Apgar III—at 10 min.

## Data Availability

The final dataset has been deposited on the Mendeley Data website (Kotfis, Katarzyna; Szylińska, Aleksandra (2022), “POC-NaVoP”, Mendeley Data, V2, doi: 10.17632/fx8394xnh4.2.) https://data.mendeley.com/datasets/fx8394xnh4/2 (accessed on 20 September 2022).

## Supplementary Materials

The following supporting information can be downloaded at: https://www.mdpi.com/article/10.3390/jcm12154978/s1, Table S1: Factors predisposing to PONV (based on the Apfel scale); Table S2: Changes in mean arterial pressure (MAP) during cesarean delivery; Table S3: Biochemical parameters for mother and neonate; Table S4: Patient satisfaction data.
