# Supplementary material for: Preoperative Oral Carbohydrate (CHO) Supplementation Is Beneficial for Clinical and Biochemical Outcomes in Patients Undergoing Elective Cesarean Delivery under Spinal Anaesthesia—A Randomized Controlled Trial
Source: J Clin Med. 2023 Jul 28;12(15):4978. doi: 10.3390/jcm12154978 (PMC10419905; doi:10.3390/jcm12154978)
Supplement: Supplementary file 1 [file jcm-12-04978-s001.zip › jcm-2468959-supplementary.pdf]

Journal of
Clinical Medicine

Table S1. Factors predisposing to PONV (based on the Apfel scale).

		Group I – CHO (n=75)		Group II - SF (n=73)		p-value
Smoking in pregnancy, n (%)	No	73	(97.33%)	67	(91.78%)	0.259
	Yes	2	(2.67%)	6	(8.22%)	
Previous PONV, n (%)	No	61	(81.33%)	56	(76.71%)	0.625
	Yes	14	(18.67%)	17	(23.29%)	
Motion sickness, n (%)	No	40	(53.33%)	38	(52.05%)	0.993
	Yes	35	(46.67%)	35	(47.95%)	
Nausea and vomiting in early pregnancy, n (%)	No	37	(49.33%)	30	(41.10%)	0.400
	Yes	38	(50.67%)	43	(58.90%)	
Nausea and vomiting in early pregnancy (0-10 scale) Me, Q1-Q3		2.00	0.00 – 6.00	4.00	0.00 – 6.00	0.432

Legend**:** CHO – oral carbohydrate drink, Me – median, Q1 – first quartile, Q3 – third quartile, n – number of patients, PONV – postoperative nausea and vomiting, , SF – Standard fasting.

Table S2. Changes in mean arterial pressure (MAP) during cesarean delivery.

Blood pressure parameters	Group I – CHO (n=75)		Group II - SF (n=73)		p-value
	Mean	SD	Mean	SD	
MAP (T0) - MAP (T1) [%]	-7.47	17.69	-3.52	11.13	0.106
MAP (T0) - MAP (T4) [%]	-23.52	15.72	-22.90	15.16	0.805
MAP (T0) - MAP (T7) [%]	-23.34	13.69	-18.36	17.25	0.050
MAP (T0) - MAP (Tfinal) [%]	-16.67	10.88	-17.85	9.72	0.489

Legend**:** CHO – oral carbohydrate drink, MAP – mean arterial pressure, n – number of patients, SD – standard deviation, SF – Standard fasting.

Table S3. Biochemical parameters for mother and neonate.

Parameters	Group I – CHO (n=75)		Group II - SF (n=73)		
	Mean	SD	Mean	SD	p-value
**Mother - plasma**					
pH	7.44	0.03	7.43	0.03	0.001
Glucose (mg/dl)	93.07	21.71	77.59	8.41	<0.001
Lactate (mmol/l)	1.62	0.49	1.21	0.46	<0.001
Plasma F2-isoPs [pg/ml]	40.15	16.89	43.78	14.91	0.049
Beta-Hydroxybutyrate (mmol/l)	1.74	0.28	1.67	0.21	0.144
HOMA-IR	10.45	8.94	2.19	1.15	<0.001
**Mother - urine**					
Urine F2-isoPs [pg/ml]	615.42	115.11	649.97	121.41	0.018
Creatinine urine [mg/ml]	0.36	0.12	0.34	0.09	0.237

Urine F2-isoPs/ Creatinine Ratio [pg/mg creatinine]	1896.12	797.12	2073.46	701.84	0.045
**Neonate – umbilical cord**					
pH	7.33	0.05	7.33	0.05	0.934
Glucose (mg/dl)	52.10	10.45	55.76	8.23	0.007
Lactate (mmol/l)	1.73	0.70	1.70	0.80	0.825

Legend: CHO – oral carbohydrate drink, n – number of patients, F2-isoPs – F2-isoprostanes, HOMA-IR – insulin resistance, Q1 – first quartile, Q3 – third quartile, SF – Standard fasting.

Table S4. Patient satisfaction data.

**Patient Satisfaction**	**Group I – CHO (n=75)**		**Group II - SF (n=73)**		**p**
Feeling of hunger before CD, mean±SD; Me	2.00	0.00 – 4.00	5.00	3.00 – 7.00	<0.001
Feeling of thirst before CD, mean±SD; Me	5.00	2.00 7.00	7.00	6.00 – 9.00	<0.001
Feeling of hunger 6 hours after CD, mean±SD; Me	3.00	2.00 – 5.00	5.00	3.00 – 6.00	0.089
Feeling of thirst 6 hours after CD, mean±SD; Me	6.00	3.00 – 8.00	7.00	4.00 – 10.00	0.059
Satisfaction from taking part in this RCT, mean±SD; Me	10.00.	10.00 – 10.00	10.00	10.00 – 10.00	0.886

All evaluations were based on a numeric rating scale from 0 to 10 points.
Legend: CHO – oral carbohydrate drink, CD – cesarean delivery**,** n – number of patients, Me – median, Q1 – first quartile, Q3 – third quartile, SF – Standard fasting.
